# The effect of repeated-sprint training on performance outcomes in youth athletes: a meta-analysis

**DOI:** 10.7717/peerj.21074

**Published:** 2026-04-15

**Authors:** Chuang Yan, Lin Shu

**Affiliations:** 1 Department of Physical Education, Tangshan Normal University, Tangshan, China; 2 Department of Music, Tangshan Normal University, Tangshan, China

**Keywords:** Repeated sprint training, Youth athletes, Meta-analysis

## Abstract

**Objective:**

This study aimed to conduct a meta-analysis to examine the effects of repeated sprint training (RST) on performance outcomes in youth athletes.

**Methods:**

The study adhered to the Preferred Reporting Items for Systematic reviews and Meta-Analyses (PRISMA) guidelines and conducted a comprehensive search of the PubMed, SportDiscus, Web of Science, SportRxiv, and ProQuest databases using specific search terms. Data extraction involved mean values and standard deviations, and methodological quality was assessed using the PEDro scale. Statistical analyses were performed using RevMan 5.3 to calculate standardized mean differences (SMD) and 95% confidence intervals. The heterogeneity of the studies was evaluated using I^2^ tests, and a sensitivity analysis was conducted when necessary.

**Result:**

The study analyzed eight studies involving 193 participants aged 13 to 18 across various sports. RST protocols varied in terms of sets, repetitions, and intensities, while the control groups (CG) followed standard curricula. Compared to CG, significant improvements were observed in 10-meter and 20-meter sprints (effect size: 1.01, 95% CI [0.52–1.50]; 0.80, [0.09, 1.52]), change-of-direction (COD) performance (effect size: 0.60, [0.14, 1.06]), and best repeated-sprint ability (RSAbest) with RST (effect size: 0.48, [0.07, 0.89]). However, sensitivity analysis indicated that the pooled 20-m sprint effect became non-significant when the study by Uthoff et al. (2020: DOI 10.1519/JSC.0000000000002914) was excluded. No significant differences were found in vertical jump performance, RSAmean, RSAdec, or maximum aerobic performance.

**Conclusion:**

RST enhances 10-m sprint, COD, and RSAbest in youth athletes. Evidence for 20-m sprint improvements should be interpreted cautiously because the pooled effect was sensitive to exclusion of a single study. RST shows limited effects on vertical jump performance, RSAmean, RSAdec, and aerobic capacity; thus, complementary training may be required. Future studies should refine RST protocols, standardize testing, and report maturation to enable maturity-stratified analyses and improve comparability across studies.

## Introduction

Repeated-sprint training (RST), characterized by its protocol of short-duration (<10 s) maximal-effort sprints followed by brief recovery intervals (<60 s) (Girard, Mendez-Villanueva & Bishop, 2011), is an time-efficient method for enhancing various physical attributes, including power output, sprint speed, aerobic capacity, and the repeated-sprints ability (RSA) (Taylor et al., 2015; Thurlow et al., 2024). These adaptations reflect improvements in both anaerobic and aerobic energy systems and can be achieved within a relatively short training period (Taylor et al., 2016).

Team and racket sports involve intermittent high-intensity actions, such as sprinting, acceleration, deceleration, and rapid changes in direction (Giles, Peeling & Reid, 2024; Stein et al., 2015; Taylor et al., 2017; Ungureanu, Lupo & Brustio, 2022), which closely align with the physiological adaptations induced by RST (Bishop, Girard & Mendez-Villanueva, 2011; Girard, Mendez-Villanueva & Bishop, 2011). RST evidence has largely been derived from adult athletes, and its effects in youth athletes remain insufficiently explored (Fernandez-Fernandez et al., 2012; Lockie et al., 2014; Lockie et al., 2012; Nebil et al., 2014; Suarez-Arrones et al., 2014). In this age group, training adaptations may differ from those observed in adults due to growth- and maturation-related factors, as described in long-term athlete development frameworks that emphasize maturational differences in trainability (Lloyd & Oliver, 2012). Given that RST targets multiple performance outcomes, including speed, power, and aerobic capacity (Glaister, 2005; Taylor et al., 2015), a comprehensive evaluation across these domains is warranted.

Previous meta-analyses examining RST effects (Taylor et al., 2015; Thurlow et al., 2024), included broader populations (predominantly adults) and non-RCT designs, and relied mainly on within-group pre–post changes without concurrent control-group (CG) comparisons. In contrast, the present analysis focuses exclusively on youth athletes, includes only RCTs, and evaluates the intervention effect using between-group pre–post change differences (RST *vs* CG). This methodological approach assessing between-group changes enables robust attribution of fitness gains to the RST intervention. By accounting for confounding variables such as normal development and other concurrent influences on performance changes over time, it strengthens causal inference regarding the efficacy of RST.

Accordingly, this study aims to meta-analyze the effects of RST on performance outcomes in youth athletes. Specifically, we quantify the pooled effects of RST on performance outcomes, including power (countermovement jump), sprint speed (sprint across various distances), change-of-direction (COD), maximal aerobic capacity, and RSA. This meta-analysis provides evidence-based insights to inform the design of effective, and developmentally appropriate RST protocols for youth athletes.

## Method

### Literature search

**Figure 1:**
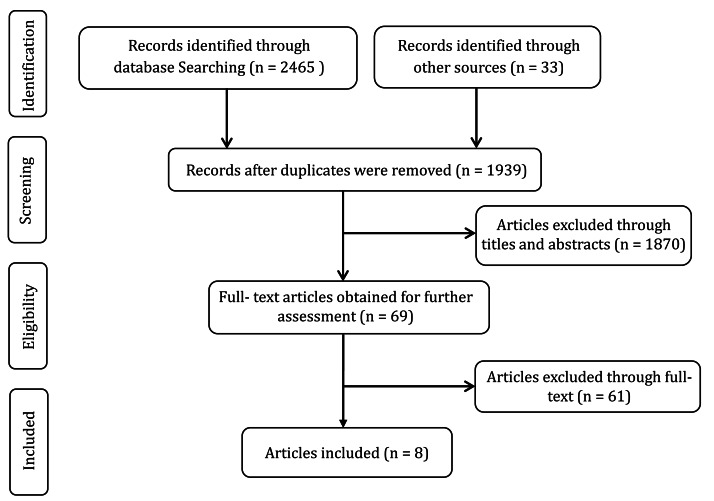
Flowchart of literature searching and screening.

The study adhered to the Preferred Reporting Items for Systematic Reviews and Meta-Analyses (PRISMA) guidelines (Moher et al., 2009) and was registered at PROSPERO (CRD420251207063). The databases PubMed, SportDiscus, Web of Science, SportRxiv, and ProQuest were comprehensively searched in October 2024 , and additional studies were identified through manual searching of the reference lists. The search terms employed were as follows: (“repeated-sprint training” OR “multiple sprint training” OR “intermittent sprint” OR “repeated sprint” OR “sprint training”) AND (“adolescent” OR “junior” OR “adolescents” OR “youth”). Two authors (C Yan, L Shu) independently screened the titles and abstracts. In cases of disagreement regarding article inclusion, a third reviewer (Lin H) acted as an arbitrator to make the final decision. Subsequently, full texts were downloaded to determine the final literature. The literature screening process is illustrated in Fig. 1.

### Study selection

The inclusion criteria for this review were as follows: (1) original experimental studies published in peer-reviewed journals or as conference proceedings, with the full text available in English; (2) studies that included both an intervention group and a CG; (3) CG continued their usual sport-specific training (*e.g.*, regular team/technical/tactical training) without additional RST; (4) the intervention training consisted of repeated RST involving maximal or near-maximal sprints with a duration of <10 s and inter-sprint intervals with a recovery period of <60 s; (5) participants were youth athletes; (6) the average age of participants ranged from 10 to 19 years; (7) the intervention lasted for a minimum of 4 weeks; (8) outcomes measured included countermovement jumps, sprints over various distances (10 m and 20 m), COD (including 20-m zigzag COD test, *T*-test, 505 COD test, and sport-specific COD tests), RSA, and maximum aerobic capacity. Sprint outcomes were extracted by distance, and COD outcomes were pooled as a single domain across eligible tests. RSA-related outcomes were selected based on evidence that RSAbest and RSAmean demonstrate acceptable reliability and validity (Bishop, Girard & Mendez-Villanueva, 2011; Glaister et al., 2007; Impellizzeri et al., 2008). Although more sensitive to variations in testing protocols and calculation methods (Bishop et al., 2001; Glaister et al., 2007), RSAdec provides complementary physiological information when interpreted alongside measures of maximal aerobic capacity.

Studies involving animal experiments and patients with specific diseases were excluded. Conference abstracts, dissertations, theses, and articles that had not undergone thorough peer review were also excluded. Studies with a mean age of participants exceeding 18.99 years were not included.

### Data extraction, and quality assessment

In the present study, mean values and standard deviations were extracted from the included studies. When information was unclear, we attempted to contact the corresponding authors *via* email; however, no additional data were obtained, and no data were estimated from graphs or imputed. The methodological quality of included studies was assessed using the Physiotherapy Evidence Database (PEDro) scale (Olivo et al., 2008), a widely used tool to evaluate the methodological quality of randomized controlled trials in rehabilitation and exercise intervention research. The criteria for PEDro scores are as follows: scores <4 are considered ‘poor’, scores of 4 to 5 are considered ‘fair’, scores of 6 to 8 are considered ‘good’, and scores of 9 to 10 are considered ‘excellent’ (Cashin & McAuley, 2020). The results of the quality assessment are shown in Table 1.

**Table 1:** Literature quality assessment form.

Study	Item											
	1	2	3	4	5	6	7	8	9	10	11	Total
Uthoff et al. (2020)	0	1	0	1	0	0	0	1	1	1	1	6
Fernandez-Fernandez et al. (2015)	0	0	0	1	0	0	0	1	1	1	1	5
Do Nascimento et al. (2015)	1	1	0	1	0	0	0	0	1	1	1	5
Haugen et al. (2014)	0	1	0	1	0	0	0	1	1	1	1	6
Sanchez-Sanchez et al. (2019)	0	1	0	1	0	0	0	1	1	1	1	6
Selmi et al. (2018)	0	1	0	1	0	0	0	1	1	1	1	6
Chtara et al. (2017)	0	1	0	1	0	0	0	1	1	1	1	6
Girard & Durussel (2015)	0	1	0	1	0	0	0	1	1	1	1	6

**Notes.**

Score of “1” was not included to total score; Across the eight included studies, allocation concealment was not reported (PEDro item 3). In addition, blinding procedures for participants, therapists, and outcome assessors were not described (PEDro items 5–7). Given the nature of exercise-based interventions, blinding of participants and therapists may be difficult to implement.

### Statistical analyses

The meta-analyses were conducted using RevMan 5.3. The mean changes and the standard deviations of the changes in the standardized mean differences (SMD) along with 95% confidence intervals were calculated. A random effects model, based on the standard errors of each included study, was employed. The mean changes were determined by subtracting the mean pre-outcome from the mean post-outcome. The standard deviation of the change was calculated as SD_change = sqrt (SD_pre^2^ + SD_post^2^ − 2 * r * SD_pre * SD_post), assuming a pre–post correlation r of 0.5, a value commonly adopted in previous studies (Currier et al., 2023; Rongwei et al., 2013; Wan et al., 2025). To assess the robustness of this assumption, sensitivity analyses were additionally conducted using alternative correlation coefficients of 0.3 and 0.7; these analyses showed that the statistical significance of the pooled results was not affected by the choice of Corr. The SMD values for highly trained athletes were categorized as follows (Rhea, 2004): 0.25 (trivial); 0.25–0.50 (small); 0.50–1.0 (moderate); and 1.0 (large). These thresholds were applied as a relative interpretative framework to facilitate comparisons between intervention and control conditions within the present sample of youth athletes. The heterogeneity of the included studies was assessed using I^2^ tests. If I^2^ ≥ 50%, a sensitivity analysis using a leave-one-out approach was performed to identify potential sources of heterogeneity by sequentially removing individual studies.

## Results

### Characteristics of the studies analyzed

Eight studies (Chtara et al., 2017; Do Nascimento et al., 2015; Fernandez-Fernandez et al., 2015; Girard & Durussel, 2015; Haugen et al., 2014; Sanchez-Sanchez et al., 2019; Selmi et al., 2018; Uthoff et al., 2020) were included, and the characteristics of the participants and the protocols of the studies are presented in Table 2. The average PEDro score was 5.75, with individual study scores ranging from 5 to 6, indicating overall fair to good methodological quality. A total of 193 participants were involved, with an average age ranging from 13 to 18 years (four studies reported ages between 13 and 15 years, while four studies reported ages between 15 and 18 years). Regarding biological maturation, only two studies assessed and reported maturity status. Chtara et al. (2017) used Tanner pubic-hair staging and reported that participants were in stages 2–3 with no between-group differences in maturity status. Uthoff et al. (2020) applied Mirwald’s maturity-offset method, reported peak height velocity (PHV)-related values with no between-group differences, and classified participants as being around PHV (mid-PHV). The remaining six studies did not quantify maturity status. Among the participants, 105 were assigned to the RST groups and 88 to the CG. The participants in the included studies represented various sports, including tennis (*n* = 2), futsal (*n* = 1), soccer (*n* = 4), and others (*n* = 1).

**Table 2:** Studies and characteristics of participants in this meta-analysis.

Study	Sample size (RST/CG), n	Age (RST/CG), years	Training experience, (years or level)	Sport	Duration/ frequency (weeks/ sessions per week)	RST protocol				CG protocol	Outcome measures
						Training structure (Sets × Reps × intensity)	Sprint (distance)	Recovery (Inter-set/ Inter-rep)	Traing mode		
Uthoff et al. (2020)	17/24	14.63/14.60	NR	NR	8/2	3 ×(1–12)×(20–45 and 50–75 and 95% Max)	45 m	NR	running/sprint	have school’s normal P.E. Curriculum	10-m, 20-m, CMJ
Fernandez-Fernandez et al. (2015)	8/8	16.9	8.0	tennis	8/2	(3–4)×(5–6)× (NR)	15–20 m	3 min/25 s	shuttle sprint	followed their normal tennis training	10 m, 20 m, 30 m, CMJ, RSAbest, RSAmean, RSAdec, VIFT
Do Nascimento et al. (2015)	10/8	16.7	≥ 4	futsal	4/2	3 × 6 ×(NR)	40 m	4 min/20 s	sprint	maintained the normal work routine	CMJ, RSAbest, RSAmean, RSAdec, PV
Haugen et al. (2014)	13/9	17	NR	soccer	9/1	1 ×(20–25)×90%	20 m	NR/60s	sprint	completed regular soccer training according to their teams’ original training plans	RSAbest, RSAmean, VO_2max_, Yo-Yo IR1, CMJ
Sanchez-Sanchez et al. (2019)	(HAF=10; LAF=10)/9	14.4–14.7/14.9	≥ 8.5 years	soccer	8/2	3 × 10 ×(NR)	18 m	4 mim/18 m	Sprint with COD	maintained regular soccer training routine	COD, RSAbest, RSAmean, RSAdec, Yo-Yo IR
Selmi et al. (2018)	15/15	17.8	elite	soccer	6/3	(2–3)×(5–6)×(NR)	30–40 m	4 min/20s	sprint	underwenting just the standard training	RSAbest, RSAdec
Chtara et al. (2017)	12/10	13.6	5.9	soccer	6/2	(2–4)×(5–6)×(100%)	20–30 m	4 min/20s	shuttle sprints	performed usual soccer training	10-m, Zigzag 20-m, RSAbest, RSAmean
Girard & Durussel (2015)	(RSTu = 5; RSTs = 5)/5	(RSTu = 12.8; RSTs = 13.6)/13.6	competitive	tennis	5/2	(3–4)×(4-6)×(NR)	10–20 m	4 min/(15–20)s	sprint; shuttle sprints	followed their normal weekly tennis training	20-m; *T*-test; CMJ; HRTT

**Notes.**

RST Repeated sprint training CG Control group RSA Repeated sprint ability (RSAbest = best performance; RSAmean = mean performance, RSAdec = performance decrement) CMJ countermovement jump COD change of direction test HRTT Hit & Run Tennis Test VIFT peak velocity from 30-15 Intermittent Fitness Test Yo-Yo IR1 Yo-Yo Intermittent Recovery Test Level 1 Zigzag 20-m 20-m zigzag change-of-direction test PV Peak velocity from Carminatti’s test -TCAR HAF high aerobic; fitness LAF low aerobic fitness RSTu RSTunidirectional RSTs RSTShuttles; 10-, 20-, 30-m = 10-, 20-, 30-m sprint NR not reported

### Protocols for RST interventions, CG, and partial tests

The average duration of intervention periods in the included studies was 6.75 weeks (range: 4 to 9 weeks), and the average frequency of interventions was 2 times per week (range: 1 to 3 times per week).

The RST protocol encompassed various sets, repetitions, and intensities (see Table 2). Among the studies reviewed, five studies utilized 3 sets (1 to 12, and 6 to 10 reps) or 3–4 sets (with 5–6 reps), while two studies employed 2–3 sets (5–6 reps) or 2–4 sets (5–6 reps). Additionally, one study implemented just 1 set (20–25 reps). In terms of intensity, three studies have reported (90%, 100%). The training modes in these studies included running, sprinting, and shuttle sprints. Conversely, five studies did not report their training modes, although it is noted that they involved sprinting or shuttle sprints. The distance for repetitions varied from 10 to 45 m, inter-set rest periods ranged from 3 to 4 min, and the duration of repetitions was between 15 to 60 s or 18 m.

The CG protocol implemented a standard physical education curriculum and adhered to conventional training methods. Six studies evaluated RSA, while four studies assessed maximum aerobic performance (Table 3).

**Table 3:** RSA and maximum aerobic performance test protocol.

Study	RSA test protocol	Maximum aerobic performance test protocol
Uthoff et al. (2020)	–	–
Fernandez-Fernandez et al. (2015)	6 repetitions of maximal 2 × 15-m shuttle sprint(∼6 s) departing every 20 s	30-15 Intermittent Fitness Test
Do Nascimento et al. (2015)	The protocol consists of eight sprints of 40 m with two changes of direction (180°) and 20 s of passive recovery period between each sprint	The progressive distance intermittent shuttle-running protocol (The progressive distance intermittent shuttle-running protocol )
Haugen et al. (2014)	12 × 20-m repeated-sprint test with sprints starting every 60 s	Yo-Yo Intermittent Recovery Level 1 test (Yo-Yo IR1)
Sanchez-Sanchez et al. (2019)	8 × 30-m linear sprints, with 25-s of low-intensity activity recovery between sprints.	Yo-Yo Intermittent Recovery test
Selmi et al. (2018)	6 ×(20 + 20-m) runs with 20 s of passive jog recover between sprints	–
Chtara et al. (2017)	6 repetitions of maximal 30 m shuttle sprints (15+15 m with 180° turns; lasting around 6 s), separated by 20 s of passive recovery	–
Girard & Durussel (2015)	–	Hit & Run Tennis Test

### The effects of RST *vs* CG on different parameters

#### Vertical jump performance

**Figure 2:**
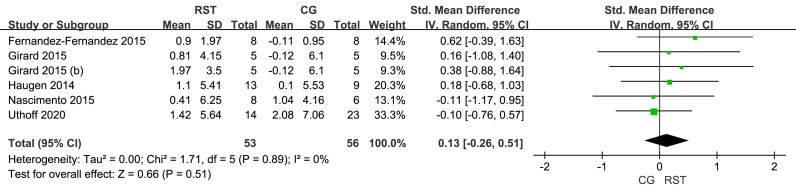
Forest plot of jump performance.

In the meta-analysis of vertical jump performance (Fig. 2), five studies were included, showing low heterogeneity among the studies (I^2^ = 0%). The overall effect size for RST and CG on CMJ was 0.13, with a 95% confidence interval spanning zero ([−0.26–0.51]), indicating no statistical significance (*p* = 0.51).

#### Sprint performance

**Figure 3:**
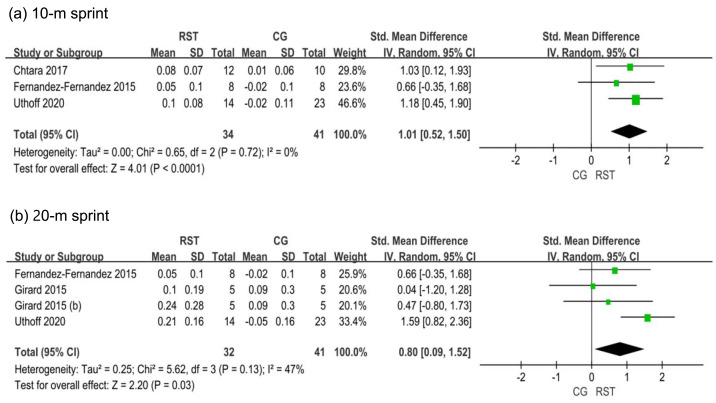
(A–B) Forest plot of sprint performance.

In the analysis of 10-meter sprint performance (Fig. 3), three studies were included, showing low heterogeneity among the studies (I^2^ = 0%). The overall effect size for RST and CG in the 10-meter sprint was 1.01, with a 95% CI [0.52–1.50], indicating statistical significance (*p* < 0.0001).

In the analysis of 20-meter sprint performance, three studies were included, showing higher heterogeneity among the studies (I^2^ = 47%; *p* = 0.13). The overall effect size for RST and CG in the 20-meter sprint was 0.80, with a 95% CI [0.09–1.52], indicating statistical significance (*p* = 0.03).

#### Change-of-direction performance

**Figure 4:**
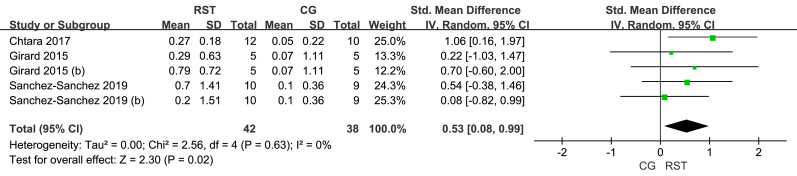
Forest plot of change of direction performance.

In the analysis of COD performance (Fig. 4), three studies were included, showing low heterogeneity among the studies (I^2^ = 0%). The overall effect size for RST and CG in the COD was 0.53, with a 95% CI [0.08–0.99], indicating statistical significance (*p* = 0.02).

#### Repeated-sprint ability

**Figure 5:**
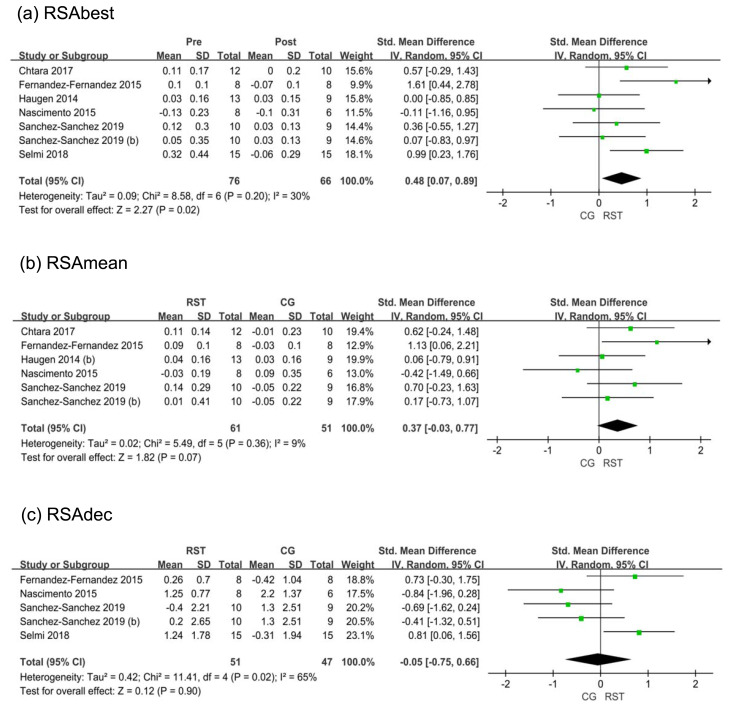
(A–C) Forest plot of repeated sprint ability.

For RSAbest (Fig. 5), a total of six studies were included, showing relatively low heterogeneity among the studies (I^2^ = 30%). The overall effect size for the RSA group compared to the CG on this metric was 0.48, with a 95% CI [0.07–0.89], indicating a statistically significant difference (*p* = 0.02).

For RSAmean, five studies were included, showing low heterogeneity among the studies (I^2^ = 9%). The overall effect size for the RSA group compared to the CG on this metric was 0.37, with a 95% CI [−0.03–0.77], indicating no statistically significant difference (*p* = 0.07).

For RSAdec, four studies were included, showing relatively high heterogeneity among the studies (I^2^ = 65%; *p* = 0.65). The overall effect size for the RSA group compared to the CG on this metric was −0.05, with a 95% CI [−0.75–0.45], indicating no statistically significant difference (*p* = 0.90).

#### Maximum aerobic performance

**Figure 6:**
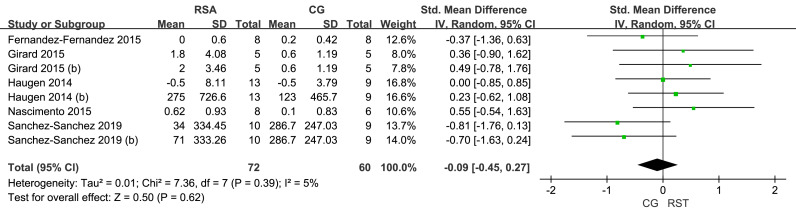
Forest plot of maximum aerobic performance.

For maximal aerobic performance (Fig. 6), five studies were included, showing very low heterogeneity among the studies (I^2^ = 5%). The overall effect size for RSA compared to the CG on the target metric was −0.09, with a 95% CI [−0.45–0.27], indicating no statistically significant difference (*p* = 0.62).

### Sensitivity analyses

Across all outcome parameters, heterogeneity approached or exceeded moderate levels for the 20-m sprint and RSAdec outcomes. Therefore, leave-one-out sensitivity analyses were conducted by sequentially excluding individual studies to explore potential sources of heterogeneity (Table 4). For the 20-m sprint outcome, exclusion of the study by Uthoff et al. (2020) reduced heterogeneity from 47% to 0%, indicating that this study was the key contributor to between-study variability. Importantly, although the pooled effect size was attenuated and no longer statistically significant after exclusion, the direction of the effect remained consistent. For the RSAdec outcome, exclusion of the study by Selmi et al. (2018) resulted in a noticeable change in the magnitude of the pooled SMD (from −0.05 to −0.30), while the result remained non-significant. This finding indicates that RSAdec outcomes are sensitive to individual study characteristics, but overall conclusions regarding the lack of a significant effect of RST on RSAdec remain robust.

**Table 4:** Sensitivity analysis results.

Parameters	Excluded study	n	SMD	95%CI	Z	*P*	Heterogeneity
20-m sprint	NA	4	0.8	0.09, 1.52	2.2	0.03	47%
	Uthoff et al. (2020)	3	0.43	−0.24, 1.09	1.25	0.21	0%
RSA_dec_	NA	5	−0.05	−0.75, 0.66	0.12	0.9	65%
	Selmi et al. (2018)	4	−0.3	−0.97, 0.37	0.88	0.38	45%

## Discussion

In this study, we included eight studies encompassing 193 participants, with an average age ranging from 13 to 18 years. The participants represented various sports, including tennis, futsal, and soccer, among others. The average PEDro score for the studies was 5.75. Participants were divided into a RST group and a CG comprising 105 and 88 individuals, respectively. The RST intervention protocol included various sets, repetitions, and intensities, while the CG followed a standard physical education curriculum and traditional training. Through the analysis of jump, sprint, COD, RSA, and maximum aerobic performance, we observed the effects of RST compared to CG across different parameters. Vertical jump performance did not show statistical significance; however, significant differences were noted in sprinting, COD, and RSAbest performance between the RST and CG. No significant difference was observed in RSAmean and RSAdec. Additionally, the analysis of maximum aerobic performance did not reveal any statistical significance.

### Vertical jump performance

Vertical jump performance was operationalized as CMJ performance based on outcomes reported in the included studies. In this meta-analysis, the vertical jump performance outcomes in the RST group did not differ significantly from the CG. Accordingly, our between-group findings suggest that RST may not directly enhance jump-related outcomes in youth athletes. The included studies consistently indicate that, compared to the CG, RST does not significantly improve jumping performance in youth athletes (Do Nascimento et al., 2015; Fernandez-Fernandez et al., 2015; Girard & Durussel, 2015; Haugen et al., 2014; Uthoff et al., 2020). In contrast, previous meta-analyses in adults have shown improvements in CMJ (Taylor et al., 2015; Thurlow et al., 2024). These meta-analyses compared within–group pre–post measures, whereas our study examines between-group change scores (RST *vs* CG). Between-group change scores (RST *vs* CG) provide a clearer estimate of the RST effect by accounting for time-related changes occurring in both groups (*e.g.*, regular training, test familiarization, and—especially in youth athletes—growth and maturation) (Vickers & Altman, 2001). In contrast, within-group pre-post changes may be inflated because they mix these time effects with random measurement variability (including regression to the mean), which can lead to over- or underestimation of the true training effect (Cuijpers et al., 2017). As a result, within-group pre-post changes may reflect both true intervention effects and statistical bias, potentially misestimating the training effect without a concurrent control group. Several factors can explain the CMJ results of this meta-analysis. Firstly, the physiological demands of RST primarily elicit neuromuscular adaptations relevant to short-distance running, including enhanced phosphocreatine synthesis, improved anaerobic energy system efficiency, and greater recruitment of motor units involved in horizontal force production (Glaister, 2005; Murray et al., 2007; Ross & Leveritt, 2001; Ross & Leveritt, 2001). These adaptations may not effectively translate into the vertical force generation required for CMJ. RST primarily involves linear sprinting with predominantly horizontal force application, whereas vertical jumping requires coordinated bilateral extension and vertical impulse production. This difference in force direction and movement demands may limit the transfer of RST adaptations to vertical jump performance. Moreover, improvements in CMJ are strongly influenced by maximal strength, eccentric braking capacity, and stretch–shortening cycle function (Komi, 2000; Möck, Hartmann & Wirth, 2023; Nishiumi et al., 2023), which are not typically emphasized or overloaded by RST alone. In youth athletes, growth and maturation may increase CMJ in both RST and CG, and the variability in neuromuscular coordination during this period (Wachholz et al., 2020) may further attenuate the observable training-specific effect of RST on vertical jump measures in between-group comparisons. Therefore, RST alone may be insufficient to improve CMJ, and may need to be combined with strength or plyometric training.

### Sprint performance

Sprint performance was interpreted as sprint time over 10 m and 20 m as reported by the included studies. This study demonstrates the efficacy of RST in improving short-distance sprint performance in youth athletes. Among the six studies included that reported sprint results, four showed significant improvements in sprint performance with RST. This finding is consistent with one study that reported significant improvements in sprint performance following RST compared with the CG (Uthoff et al., 2020). In this meta-analysis, RST exhibited a large effect size advantage over the CG in the 10-meter sprint and a moderate effect size advantage in the 20-meter sprint, based on commonly used effect size interpretation thresholds. It should be noted that the classifications of effect size were used as a relative interpretative framework for comparisons within the present sample of youth athletes and do not necessarily indicate clinical meaningfulness. Mechanistically, RST induces fatigue in athletes, and this fatigue typically manifests later in the training protocol. The RST protocols in the studies included distances ranging from 10 to 45 m, which primarily target acceleration and short sprint performance. Athletes complete the initial sprints with ample energy and vigor, and this repeated exposure over time may lead to substantial improvements in short sprint performance. Such high-intensity short sprints may enhance metabolic, muscular, and neural adaptations (Murray et al., 2007; Ross & Leveritt, 2001; Ross & Leveritt, 2001), thereby improving acceleration and maximum speed. Regarding the 20-meter sprint outcome, Uthoff et al. (2020) exerted a notable influence on the pooled results. Upon its exclusion, the effect size for the 20-meter sprint decreased to 0.43, indicating that this study may be an important contributing factor; however, it met the inclusion criteria and could not be disregarded. Excluding Uthoff et al. (2020) also reduced heterogeneity from 47% to 0% but rendered the pooled 20-m sprint effect non-significant, indicating that this finding should be interpreted cautiously. Notably, Uthoff et al. (2020) was the only study that used exclusively linear sprints as the RST protocol, which may partly explain its larger contribution to the pooled sprint effect. Collectively, these findings suggest that study-specific characteristics, particularly RST protocol design, contributed to the observed between-study heterogeneity.

### Change-of-direction performance

In this meta-analysis, COD performance was defined as COD test completion time (s) as reported in each study, with protocols varying across tests. The statistically significant improvement and moderate pooled effect size suggest a potential benefit of RST for COD performance. However, effect-size magnitudes are interpreted using conventional thresholds and should be viewed as relative. Across the three included studies that reported COD outcomes, only one indicated a significant enhancement in COD performance, and this study implemented shuttle sprints as the RST protocol. Consistent with prior trials, Chtara et al. (2017) reported greater improvements in COD performance following RST compared with the CG, although the overall COD evidence base remains small in the present meta-analysis. The efficacy of RST for optimizing COD may be limited compared with interventions that more directly target COD-specific determinants, particularly when considering the multifactorial nature of COD ability in youth athletes. COD performance depends on several trainable components, including strength and eccentric capacity (Spiteri et al., 2015; Spiteri et al., 2014), rate of force development (Yamashita, Sato & Mishima, 2024; Young, James & Montgomery, 2002), and technical execution (Menezes et al., 2024), as well as cognitive demands (Sheppard & Young, 2006). Therefore, enhancements in COD performance may arise from specific training that increases familiarity and movement efficiency, and all included interventions incorporated some form of COD exposure, which may have contributed to the observed changes.

In addition, biological maturation may be associated with improvements in both sprint and COD performance in youth athletes (Baker et al., 2025; Giuriato et al., 2023). However, because most included studies did not assess or report maturity status, it was not possible to determine whether maturation moderated the effects of RST on COD and sprint performance. Accordingly, the potential confounding influence of maturation should be interpreted cautiously. Although RST can improve COD performance, it may not necessarily enhance the physical attributes or cognitive demands associated with changing directions, which could account for the relatively smaller effect size compared to sprint performance. It is also important to note that improvements in COD performance may be a byproduct of enhanced sprint performance.

### Repeated-sprint ability

Our findings suggest that while RST is effective at improving the best sprint times during repeated-sprint tasks, it does not significantly improve the ability to maintain performance (*i.e.,* RSAmean and RSAdec, as reported) across multiple sprints. The results of this meta-analysis on RSA are consistent with previous literature, highlighting the effectiveness of RST in enhancing RSAbest performance (Fernandez-Fernandez et al., 2015; Selmi et al., 2018). Enhancements in RSAbest are particularly important for sports that require high-intensity intermittent activities, such as soccer, tennis, and rugby. RSAbest represents the fastest sprint performance achieved during repeated-sprint tasks and reflects athletes’ maximal sprinting capability. From a physiological perspective, improvements in RSAbest likely reflect enhanced initial sprint performance, which is primarily supported by rapid phosphagen energy provision and neuromuscular factors during the early repetitions of repeated-sprint tasks (Ross & Leveritt, 2001; Ross, Leveritt & Riek, 2001). In youth athletes, RSA performance across repetitions appears closely linked to the pattern of mechanical output, suggesting an important role of sprint mechanical capabilities and neuromuscular ability in influencing repeated-sprint performance (Glaude-Roy et al., 2023; Runacres, Mackintosh & McNarry, 2023). Accordingly, these youth-specific responses may help explain why RST more consistently improves best sprint performance than performance decrement across sprints. From a training and testing perspective, the RSA test protocols adopted in each included study were closely aligned with their corresponding RST intervention designs, indicating a high degree of specificity between the training stimulus and the performance assessment (Buchheit, 2012). This observed improvement may be attributable to adaptations arising from the close correspondence between the RST protocols and the RSA testing procedures.

### RSAdec and maximum aerobic ability

This meta-analysis revealed that RSAdec and maximum aerobic ability did not show significant improvements. Among the studies included, only Selmi et al. (2018) reported a significant enhancement in RSAdec following RST, whereas the remaining studies reported no notable changes in RSAdec or maximum aerobic performance. Although previous adult meta-analyses have indicated that RST enhances performance on the Yo-Yo Intermittent Recovery Test (Thurlow et al., 2024), the aerobic outcomes in the present analysis were assessed using different intermittent running tests, which may limit direct comparability. Overall, pooled effects for these outcomes were small. Although studies suggest that RSA is dependent on the ability to recover between repeated high-intensity efforts, which is supported by aerobic recovery processes and metabolite regulation (*e.g.*, lactate/H+ handling) (Ratel et al., 2002), the present meta-analysis showed improvements in sprint and COD performance, whereas RSAdec and maximum aerobic ability did not change significantly. This pattern indicates that observed RSA-related gains may be more closely linked to sprint- and COD-related adaptations than to aerobic improvements, although this interpretation should be viewed cautiously. The lack of improvement in maximum aerobic ability aligns with the LTAD model, which posits that adolescence may be a less sensitive period for endurance development (Varghese, Ruparell & LaBella, 2022).

Physiologically, RSAdec and maximum aerobic performance reflect the ability to sustain repeated high-intensity efforts and recover between bouts. In youth athletes, RSA is influenced by performance decline and recovery across sprints, and is supported by aerobic recovery processes as well as metabolite regulation (*e.g.*, lactate/H+ handling) (Ratel et al., 2002); as a high-intensity exercise, RST imposes substantial acute metabolic and mechanical demands (Baena-Raya et al., 2025; Runacres, Mackintosh & McNarry, 2023), and youth HIIT studies suggest such stimuli may improve aerobic-related outcomes (Engel et al., 2018). The lack of significant improvement in RSAdec and maximum aerobic ability may also be attributed to the RST protocols used in the included studies, which may not have been optimally designed for enhancing aerobic recovery processes and metabolite regulation. Protocols that incorporate longer running distances, greater total sprint volumes, or larger work-to-rest ratios may have a greater potential to improve RSAdec and maximum aerobic capacity. In addition, because relatively long recoveries are often prescribed (Thurlow et al., 2023) to preserve sprint quality, the accumulated time under sustained aerobic stress may be insufficient, thereby limiting improvements in maximal aerobic performance. It is also possible that the training dose of RST in the included studies was insufficient to elicit meaningful adaptations in resistance to performance decrement and recovery between bouts, as well as aerobic recovery capacity, given that intervention duration and weekly frequency were relatively modest in several studies and training intensity was not consistently reported. Therefore, improving RSAdec and aerobic capacity may require RST to be supplemented with additional aerobic-oriented training. Additionally, endurance-related outcomes may show smaller changes if participants already have a relatively high sport-derived aerobic base, leaving limited room for further improvement.

Furthermore, differences in the calculation formulas of RSA testing protocols may have a greater impact on RSAdec results compared to RSA mean and RSA best. Sensitivity analysis indicated that removal of the study by Selmi et al. (2018) resulted in a change in the pooled standardized mean difference for RSAdec from −0.05 to −0.30, accompanied by a reduction in heterogeneity. However, this adjustment did not alter the level of statistical significance. Given that the training interventions across studies contributing to RSAdec were relatively comparable, this variation is more likely attributable to inconsistencies in the calculation methods used for RSAdec across studies. RSAdec was nevertheless retained as an outcome of interest because the overall conclusion remained robust, namely that repeated-sprint training does not significantly improve RSAdec in youth athletes.

### Limitations and implications for future research

We acknowledge several limitations in this meta-analysis. First, effect sizes were interpreted with caution because commonly used thresholds are largely based on adult athletic populations and may not directly apply to youth athletes. Second, the meta-analysis protocol was registered retrospectively after the literature search and data extraction, which limited prospective pre-specification of some methodological decisions. Third, the moderate methodological quality of the included studies, as reflected by PEDro scores, may reduce confidence in the pooled estimates. Fourth, the small number of studies available for each outcome limited statistical power and prevented further subgroup or sensitivity analyses. In addition, biological maturation could not be examined as a moderator because only two included studies reported maturity status, while the remaining studies provided no such information. Methodological heterogeneity may also have influenced the pooled results, including variability in training intervention characteristics across studies and differences in RSAdec calculation methods; therefore, the findings should be interpreted with caution. Moreover, training prescription variables (*e.g.*, intensity and recovery structure/duration) were not reported consistently across studies, which may limit precise replication of the included RST protocols. Finally, maximal aerobic performance was assessed using different protocols across studies (*e.g.*, Yo-Yo IR1, VIFT, and TCAR), which may introduce additional variability and reduce comparability of aerobic outcomes. Accordingly, aerobic-performance findings should be interpreted cautiously.

The results of this meta-analysis have implications for the design of training programs for youth athletes. Athletes in this age range, may respond differently to training across fitness components because they are still growing and maturing. Therefore, future studies should, where feasible, assess and report biological maturation status (*e.g.*, maturity offset or PHV-related indicators) to better interpret training responsiveness and enable stratified analyses. Furthermore, RST may be best considered one element of a comprehensive sport-performance approach, aligning with contemporary integrated training paradigms such as the joint-by-joint approach advocated by Dhahbi, Materne & Chamari (2025). This perspective is particularly relevant for youth athletes, who are still developing fundamental neuromuscular coordination and sport performance. Therefore, practitioners may consider integrating RST with complementary training elements (*e.g.*, strength, plyometrics, and agility training) instead of relying on isolated RST alone, to support performance gains, injury risk reduction, and long-term athletic development. RST may not necessarily be the optimal method for improving vertical jump, RSAdec, and maximum aerobic ability in youth athletes. Future research could modify RST protocols by adjusting work-to-rest ratios, sprint volumes, and rest intervals to determine if these changes can more effectively enhance vertical explosive power and fatigue resistance in repetitive actions. Future studies should compare RST with other intermittent training methods using matched training loads to clarify its relative effectiveness. Methodologically, future research should propose a standardized testing protocol for RSA assessment to improve comparability across studies.

## Conclusion

RST can enhance specific performance metrics in youth athletes; in particular, the available evidence suggests improvements in 10-meter sprint performance, COD performance, and RSAbest. Evidence for 20-meter sprint improvements should be interpreted cautiously, as the pooled effect became non-significant in sensitivity analysis. However, RST may not be the optimal training method for CMJ, RSAmean, RSAdec and maximum aerobic capacity. Youth athletes may be especially responsive to speed- and COD-related adaptations, whereas endurance development may be less pronounced during this period; however, this interpretation should be treated cautiously because most included studies did not report biological maturation status. Overall, RST is a useful method to enhance sprint and COD performance; its limited impact on other metrics highlights the need for complementary training methods, such as resistance or plyometric exercises, to optimize overall athletic development. Future research should refine RST protocols, adopt standardized testing methods, and assess and report biological maturation status (*e.g.*, maturity offset/PHV) to enable maturity-stratified analyses and improve comparability across studies.

##  Supplemental Information

10.7717/peerj.21074/supp-1 Supplemental Information 1 Raw data

10.7717/peerj.21074/supp-2 Supplemental Information 2 PRISMA checklist

10.7717/peerj.21074/supp-3 Supplemental Information 3 Rationale

## Additional Information and Declarations

### Data Availability

The following information was supplied regarding data availability: This is a Systematic Review/Meta-analysis.
