# Supplementary material for: The effect of repeated-sprint training on performance outcomes in youth athletes: a meta-analysis
Source: PeerJ. 2026 Apr 15;14:e21074. doi: 10.7717/peerj.21074 (PMC13091579; doi:10.7717/peerj.21074)
Supplement: Supplemental Information 3 [file peerj-14-21074-s003.docx]

1. **The rationale for conducting the systematic review/meta-analysis.**

Team and racket sports are characterized by a unique combination of prolonged low-intensity activities interspersed with bursts of high-intensity actions, such as sprinting, acceleration, deceleration, and rapid changes in direction(Giles et al., 2024; Stein et al., 2015; Taylor et al., 2017; Ungureanu et al., 2022). These demands closely align with the physiological adaptations promoted by repeated sprint training (RST) (Bishop et al., 2011; Girard et al., 2011). While research supporting the benefits of RST continues to grow, it has primarily focused on adult athletes (Fernandez-Fernandez et al., 2012; Lockie et al., 2014; Lockie et al., 2012; Nebil et al., 2014; Suarez-Arrones et al., 2014). Consequently, there is a significant gap in understanding its effects on adolescent populations.

Although meta-analyses have examined the effects of RST on fitness outcomes, none specifically target adolescent athletes. The adolescent phase is a critical period for physical maturation and athletic development (Ford et al., 2010; John et al., 2019; Perroni et al., 2018; Towlson et al., 2018). Crucially, training responses can differ between adults and adolescents, highlighting the need for dedicated investigation into the impact of RST on fitness outcomes in young athletes. Investigating how RST influences this demographic is therefore essential for refining tailored training methodologies. Furthermore, given that RST targets multifaceted performance outcomes—ranging from speed and power to aerobic capacity—a comprehensive evaluation of its effectiveness across these domains is necessary.

1. **The contribution that it makes to knowledge in light of previously published related reports, including other meta-analyses and systematic reviews.**

The present meta-analysis specifically investigates the effects of RST on adolescent athletes, addressing a gap in the existing literature. Previous meta-analyses examining RST effects, which included broader populations (predominantly adults), were limited by not exclusively incorporating randomized controlled trials (RCTs) and by relying solely on pre-post comparisons within the intervention group without a control group comparison. In contrast, the present analysis focuses exclusively on adolescents, incorporates only RCT designs, and crucially compares the pre-post changes in the RST group relative to those observed in a concurrent control group. This methodological approach, which assesses between-group change differences, enables a more robust attribution of observed fitness gains to the RST intervention. By accounting for potential confounding variables such as normal maturation or other concurrent influences that may also contribute to performance changes over time, it enhances the causal inference regarding the efficacy of RST.

Furthermore, The results of this meta-analysis have significant implications for the design of training programs for adolescent athletes. Adolescents, being in a unique growth phase, have varying requirements for the development of different physical qualities. The beneficial outcomes demonstrated in this study specifically target the sensitive physical qualities developed during adolescence, indicating the applicability of RST for physical training in this population. While RST is effective in improving 10-m and 20-m sprint performances, COD performance, RSAbest, and RSAmean, coaches and practitioners should consider incorporating complementary training methods to enhance these metrics, such as resistance training, weightlifting, and plyometric training. RST may not necessarily be the optimal method for improving vertical jump, RSA_dec_, and maximum aerobic ability in adolescents.

Bishop D, Girard O, and Mendez-Villanueva A. 2011. Repeated-Sprint Ability — Part II. *Sports Medicine* 41:741-756. 10.2165/11590560-000000000-00000

Fernandez-Fernandez J, Zimek R, Wiewelhove T, and Ferrauti A. 2012. High-intensity interval training vs. repeated-sprint training in tennis. *J Strength Cond Res* 26:53-62. 10.1519/JSC.0b013e318220b4ff

Ford KR, Myer GD, and Hewett TE. 2010. Longitudinal effects of maturation on lower extremity joint stiffness in adolescent athletes. *Am J Sports Med* 38:1829-1837. 10.1177/0363546510367425

Giles B, Peeling P, and Reid M. 2024. Quantifying Change of Direction Movement Demands in Professional Tennis Matchplay: An Analysis From the Australian Open Grand Slam. *J Strength Cond Res* 38:517-525. 10.1519/jsc.0000000000003937

Girard O, Mendez-Villanueva A, and Bishop D. 2011. Repeated-Sprint Ability — Part I: Factors Contributing to Fatigue. *Sports Medicine* 41:673-694. 10.2165/11590550-000000000-00000

John C, Rahlf AL, Hamacher D, and Zech A. 2019. Influence of biological maturity on static and dynamic postural control among male youth soccer players. *Gait Posture* 68:18-22. 10.1016/j.gaitpost.2018.10.036

Lockie RG, Murphy AJ, Callaghan SJ, and Jeffriess MD. 2014. Effects of sprint and plyometrics training on field sport acceleration technique. *J Strength Cond Res* 28:1790-1801. 10.1519/jsc.0000000000000297

Lockie RG, Murphy AJ, Schultz AB, Knight TJ, and Janse de Jonge XA. 2012. The effects of different speed training protocols on sprint acceleration kinematics and muscle strength and power in field sport athletes. *J Strength Cond Res* 26:1539-1550. 10.1519/JSC.0b013e318234e8a0

Nebil G, Zouhair F, Hatem B, Hamza M, Zouhair T, Roy S, and Ezdine B. 2014. Effect of optimal cycling repeated-sprint combined with classical training on peak leg power in female soccer players. *Isokinetics and Exercise Science* 22:69-76.

Perroni F, Pintus A, Frandino M, Guidetti L, and Baldari C. 2018. Relationship Among Repeated Sprint Ability, Chronological Age, and Puberty in Young Soccer Players. *J Strength Cond Res* 32:364-371. 10.1519/jsc.0000000000001799

Stein JG, Gabbett TJ, Townshend AD, and Dawson BT. 2015. Physical qualities and activity profiles of sub-elite and recreational Australian football players. *J Sci Med Sport* 18:742-747. 10.1016/j.jsams.2014.10.008

Suarez-Arrones L, Tous-Fajardo J, Núñez J, Gonzalo-Skok O, Gálvez J, and Mendez-Villanueva A. 2014. Concurrent repeated-sprint and resistance training with superimposed vibrations in rugby players. *Int J Sports Physiol Perform* 9:667-673. 10.1123/ijspp.2013-0238

Taylor JB, Wright AA, Dischiavi SL, Townsend MA, and Marmon AR. 2017. Activity Demands During Multi-Directional Team Sports: A Systematic Review. *Sports Med* 47:2533-2551. 10.1007/s40279-017-0772-5

Towlson C, Cobley S, Parkin G, and Lovell R. 2018. When does the influence of maturation on anthropometric and physical fitness characteristics increase and subside? *Scand J Med Sci Sports* 28:1946-1955. 10.1111/sms.13198

Ungureanu AN, Lupo C, and Brustio PR. 2022. Padel Match Analysis: Notational and Time-Motion Analysis during Official Italian Sub-Elite Competitions. *Int J Environ Res Public Health* 19. 10.3390/ijerph19148386
